# What is Menière’s disease? A contemporary re-evaluation of endolymphatic hydrops

**DOI:** 10.1007/s00415-015-7930-1

**Published:** 2016-04-15

**Authors:** R. Gürkov, I. Pyykö, J. Zou, E. Kentala

**Affiliations:** Department of Otorhinolaryngology Head and Neck Surgery, University of Munich, Marchioninistr. 15, 81377 Munich, Germany; German Centre for Vertigo and Balance Disorder, University of Munich, Marchioninistr. 15, 81377 Munich, Germany; Hearing and Balance Research Unit, Otolaryngology, School of Medicine, University of Tampere, 33520 Tampere, Finland; Department of Otorhinolaryngology, Helsinki University Central Hospital, Haartmaninkatu 4E, 00290 Helsinki, Finland

**Keywords:** Menière’s disease, Endolymphatic hydrops, Magnetic resonance imaging, Diagnosis, Classification

## Abstract

Menière’s disease is a chronic condition with a prevalence of 200–500 per 100,000 and characterized by episodic attacks of vertigo, fluctuating hearing loss, tinnitus, aural pressure and a progressive loss of audiovestibular functions. Over 150 years ago, Prosper Menière was the first to recognize the inner ear as the site of lesion for this clinical syndrome. Over 75 years ago, endolymphatic hydrops was discovered as the pathologic correlate of Menière’s disease. However, this pathologic finding could be ascertained only in post-mortem histologic studies. Due to this diagnostic dilemma and the variable manifestation of the various audiovestibular symptoms, diagnostic classification systems based on clinical findings have been repeatedly modified and have not been uniformly used in scientific publications on Menière’s disease. Furthermore, the higher level measures of impact on quality of life such as vitality and social participation have been neglected hitherto. Recent developments of high-resolution MR imaging of the inner ear have now enabled us to visualize in vivo endolymphatic hydrops in patients with suspected Menière’s disease. In this review, we summarize the existing knowledge from temporal bone histologic studies and from the emerging evidence on imaging-based evaluation of patients with suspected Menière’s disease. These indicate that endolymphatic hydrops is responsible not only for the full-blown clinical triad of simultaneous attacks of auditory and vestibular dysfunction, but also for other clinical presentations such as “vestibular” and “cochlear Menière’s disease”. As a consequence, we propose a new terminology which is based on symptomatic and imaging characteristics of these clinical entities to clarify and simplify their diagnostic classification.

## Introduction

Prosper Menière reported in 1861 that vertigo, balance and hearing diseases reflected a lesion of the inner ear [1]. Previously, dizziness and balance diseases had been attributed to “apoplectiform cerebral congestion”, and the anatomical structures of the inner ear were only considered with respect to sound perception. As a director of the first school for the deaf-mute in Paris, Prosper Menière undoubtedly saw many patients with the combination of deafness and vertigo. However, the role of the inner ear in maintaining balance and orientation was largely unknown at that time. The combination of his clinical experience with this patient group and his knowledge of Flourens’ seminal work on the effects of semicircular canal ablation in pigeons allowed him to recognize the inner ear as the site of lesion.

The cardinal symptoms of Menière’s disease (MD) form a disease entity consisting of episodic vertigo, fluctuant hearing loss and tinnitus. The patients also complain of fullness in the ear, gait problems, postural instability, drop attacks and nausea. MD is a chronic illness affecting about 190 per 100,000 patients in a US health claims database, but in population-based studies a prevalence of as high as 513/100,000 has been reported [2]. In 1937, the discovery of endolymphatic hydrops (EH) in human temporal bones by British and Japanese researchers [3, 4] revealed the pathologic counterpart of the clinical syndrome described by Prosper Menière. EH is a distension of the endolymphatic space of the inner ear into areas that are normally occupied by the perilymphatic space. It most often occurs in the cochlear duct and the sacculus but may also involve the utricle and the semicircular canals [5]. Analysis of temporal bone specimens has shown variability of the presence of EH [6] and Salt and Plontke [7] questioned whether the presence of post-mortem EH is either essential or specific to MD. Recent developments of gadolinium chelate (GdC)-enhanced MRI after transtympanic injection of the contrast agent provide a tool for separately visualizing endolymphatic and perilymphatic spaces with gadolinium chelate (GdC) as the contrast agent [8]. With these new imaging techniques, EH can be demonstrated in vivo and can be used to confirm the diagnosis.

In this article, we shall summarize important recent developments in the evaluation of EH in MD and discuss the future impact of these insights on its classification.

## Evidence from human temporal bone studies

Morita et al. [9] examined 53 temporal bones and quantified endolymphatic hydrops in patients with Menière’s disease: the collective endolymphatic volume of the cochlear duct, saccule and utricle amounted to 64 µl in comparison to 20 µl in healthy subjects. Therefore, the very tightly controlled minuscule endolymphatic fluid space of the inner ear is enlarged by more than 200 % in MD! Of all the hitherto known pathologic changes in MD patients, this change clearly has the highest magnitude.

However, in order to obtain clues that help us to understand (1) what is the pathophysiologic consequence of EH? and (2) what events lead to the development of EH?, other pathologic changes that are found in MD patients have to be considered as well.

Nageris et al. [10] described a related phenomenon: the displacement of the basilar membrane towards the scala tympani in the apical cochlear regions. In MD patients’ temporal bones, there was a significant correlation between the severity of EH and the basilar membrane displacement. The reason why this phenomenon was found only in the apical portion of the cochlea is probably the larger width and higher elasticity of the basilar membrane compared to the basal cochlear regions and the lack of a supporting bony structure of the apical Lamina spiralis. This feature is a consequence of EH that has severe functional consequences, since the basilar membrane and its specific biomechanic properties are an essential part of the mechanoelectrical transfer function of the hearing system.

Other morphologic changes that have been observed in MD give not such a clear picture. Unfortunately, the research on inner ear pathology has not been systematically promoted for a long time. Until 1995, examinations of only 100 cases of MD have been published worldwide, and many of those were based on insufficient clinical information. Often, a vestibular fibrosis is observed, with the formation of band-like fibrous structures. These may create a connection between the stapes footplate and the utricular macula, which in turn could be an explanation for the Hennebert sign (occurrence of vertigo when static pressure is applied to the ear canal) [11]. Within the endolymphatic sac (ELS), an increased amount of intraluminal precipitate, consisting of glycoproteins secreted by the ELS, has been demonstrated [12]. Furthermore, ultrastructural evidence suggests that glycoprotein synthesis in the rough endoplasmatic reticulum and Golgi complexes is hyperactive in MD patients [13]. Accumulation of Glycoproteins in the ELS could by its osmotic effect interfere with inner ear homeostasis and contribute to EH formation.

Electron microscopy studies revealed minimal changes of the cochlear hair cells: fusion of stereocilia and displacement of outer hair cells towards the basilar membrane, with loss of contact to the cuticular plate [14, 15], a phenomenon, which by itself may disable the cochlear amplifier function of the outer hair cells and, therefore, lead to hearing loss.

Further findings are a neural fiber loss in the spiral osseus lamina [16] and a reduced number of afferent nerve endings and afferent synapses at the basis of inner and outer hair cells [15]. Tsuji et al. could show a significant reduction of type II hair cells in all five vestibular end organs and of vestibular ganglion neurons [17]. Another recent study on 39 temporal bones found a marked loss of neurons of the spiral ganglion, in both the ipsilateral and contralateral ear in patients with unilateral MD [18]. A similar magnitude of loss of cochlear inner and outer hair cells was found (about 70 %). The stria vascularis, which can be regarded as the “power plant” of inner ear homeostasis, was found to be atrophic (reduced in area) and suffering from a reduced blood vessel density [19].

In summary, besides EH, several degenerative changes could be observed in the audiovestibular periphery of MD patients, especially in the afferent vestibular and cochlear ganglia and nerves. However, these findings do not yet allow for definitive conclusions on the sequence of pathophysiologic events during the development and progress of the disease.

## Relationship between histologically proven EH and clinical definite Menière’s disease

Despite the development of several animal models of EH, none of these models displays the typical phenotype observed in human MD patients: paroxysmal audiovestibular events plus chronic-progressive loss of inner ear functions. Therefore, we shall concentrate on evidence from human patients when considering the relationship between EH and clinical MD in patients.

In a recent review, Foster et al. [20] analyzed all published articles that have reported on temporal bones with EH and/or on temporal bones of patients with clinically suspected MD. This resulted in a total of 3707 temporal bone specimens. Of these, 165 cases had been reported to fulfill the AAO-HNS 1995 criteria. Two of these studies were specifically designed to explore the relationship of EH to MD that meets the AAO-HNS 1995 criteria, and found EH in 100 % of MD cases [6, 21]. 163 of the temporal bones from definite MD patients in this review (98.8 %) had EH in at least one ear. Only two of 165 cases had been classified as MD without EH, and these cases were mentioned incidentally in a single study of strial changes in the contralateral ear of MD patients. Foster et al. communicated with the authors of that study [18] and report that both cases were diagnosed before the AAO-HNS 1995 criteria, and that their clinical presentation was not described so it is impossible to verify whether they fulfilled the AAO-HNS criteria during their lifetime. None of these cases can be used to refute the primary finding of the Merchant study that EH and MD are found in association with 100 % of cases when the current definition of MD is strictly applied.

This indicates that it is virtually certain that EH is present in at least 1 temporal bone in a person who meets current MD criteria. The authors conclude that EH is unlikely to be just an epiphenomenon of MD, because the association is perfect: every case with MD according to the AAO-HNS criteria showed EH. It seems, therefore, that EH is necessary but not sufficient for the display of the full symptom triad of MD.

## Diagnostic criteria: evolution of the current criteria for assessment of Menière’s disease

Symptom-based classification methods have been used to make the diagnosis [22]. In the diagnostic work up, mainly vertigo character and type, associated hearing loss and tinnitus or aural fullness are taken into consideration. Indeed, in a taxonomic investigation of patients with vertigo, after exclusion of neurological and middle ear conditions, head trauma and ototoxicity, Hinchcliffe [23] found that those with ‘classical’ Menière’s disease (meeting the ‘‘definite MD’ definition below) fell in a single nosological entity with all the other cases of vertigo. He later argued that MD included ‘formes frustes’, where the triad of symptoms is not complete [24]. Diagnostically confirmed cases represent only a limited proportion of individuals with the disease, as reflected in the variability between prevalence studies [2, 25].

The nomenclature of “cochlear” or “vestibular” MD was coined by the American Academy of Otolaryngology-Head and Neck Surgery (AAO-HNS) in 1972 [26] and was abandoned with the 1985 [27] and 1995 [22] updates of the AAO-HNS criteria as there was insufficient evidence that these mono-symptomatic diseases share the same pathophysiology with MD. The revised AAO-HNS criteria [22] define ‘Possible MD’ as episodic vertigo or fluctuating hearing loss. ‘Probable MD’ consists of one attack of rotatory vertigo lasting at least 20 min together with tinnitus and documented hearing loss. ‘Definite MD’ consists of two or more spontaneous episodes of vertigo 20 min or longer with tinnitus and documented hearing loss. ‘Certain MD’ is diagnosed by additional histological verification of EH in the inner ear. To define the condition clinically, the existing AAO-HNS classification is often unhelpful as the latency of joint presentation of the cardinal complaints may take up to 10 years [28]. General practitioners, otolaryngologists and audio-vestibular physicians face a challenge in making the diagnosis of MD. The symptoms can be variable, occur over different time spans and the hearing loss can recover before audiometric measurements are made [22].

Recently, the Classification Committee of the Bárány Society formulated diagnostic criteria for MD jointly with several national and international organizations [29]. The classification includes two categories: definite MD and probable MD. The diagnosis of definite MD is based on clinical criteria and requires the observation of an episodic vertigo syndrome associated with low- to medium-frequency sensorineural hearing loss and fluctuating aural symptoms (hearing, tinnitus and/or fullness) in the affected ear. Duration of vertigo episodes is limited to a period between 20 min and 12 h. Probable MD is a broader concept defined by episodic vestibular symptoms (vertigo or dizziness) associated with fluctuating aural symptoms occurring in a period from 20 min to 24 h. These definitions unfortunately do not help the clinician in defining MD. One interesting difference is that the proposed definition does not include endolymphatic hydrops that was the original finding in the disease.

Recent novel imaging methods have made it possible to visualize EH with gadolinium contrasted 3T MRI. The AAO-HNS (1995) criteria [22] include EH as landmark to define certain MD. Recently, Nakashima et al. [30] suggested that the inner ear of all patients with suspected MD should be imaged and the classification as definite MD should include MRI evidence of EH. The authors propose that also monosymptomatic ears with EH could be treated as MD in the same way as in the 1972 AAO-HNS classification, which recognized vestibular MD and cochlear MD as one disease entity among the umbrella of MD [26]. Supporting this idea, Pyykkö et al. [28] reported that in about 20 % of the patients with MD it can take more than 5 years and in 10 % even more than 10 years before cochlear and vestibular symptoms will coincide.

To conclude, we propose that diagnosis of MD should be based on the presence of EH in addition to symptoms and that also monosymptomatic patients with EH be regarded as ‘certain’ MD cases. MRI investigations should be made more frequently in assessing MD than hitherto.

## Clinical features of Menière’s disease

Although the cardinal symptoms of vertigo, hearing loss and tinnitus are generally well acknowledged by physicians, MD patients often complain also of pressure or fullness in the ear, gait problems, postural instability, Tumarkin attacks and nausea [31, 32]. To determine the severity of the impact on the patients’ quality of life, several symptom-specific scoring instruments have been developed. Such rating scales are, e.g., the Hearing Disability and Handicap Scale [33, 34], the Vertigo Handicap Index [35], and the International Tinnitus Inventory [36]. A MD-specific indicator is the MD Patient Oriented Severity Index (MDPOSI) [37]. Some of these have been developed to evaluate changes in the natural course or therapeutic effects, such as MDPOSI. The symptom-specific instruments seem to more accurately reflect changes in control of vertigo in MD over time than do, e.g., general Quality of Life (QoL) instruments [32]. These indicators seem to be capable of describing changes in the activity of the disease and are used in the validation of the efficacy of the treatment [38, 39]. In addition, it seems that personal trait measured as sense of coherence, attitude and mood are important determinants for the impact of MD [32, 39, 40]. Stephens et al. [41] pointed out that anxiety, as a mood disorder, will reflect expectations, environmental demands and attitudes. They showed that the level of anxiety correlated with the Sense of Coherence [40].

However, the personal factors, uncertainty of life and environmental factors have not been included in the different complaint-oriented impact classifications. In this regard, the International Classification of Function group (ICF, WHO 2001) [42] has developed a system encompassing many different aspects of the disease, which can be used as explanatory framework. This framework allows a better understanding of the impact of the illness and what consequences it has on general well-being and, therefore, may help to alleviate these impacts. Social participation which is included in the ICF is a vital part of life in human behavior that forms the core construct of the level of activities enabling goal-directed behavior. When establishing treatment strategies, ICF includes two most important additional topics: own attitudes and personal contextual factors, as pointed out by Wade [43].

In MD, ICF brings in some important elements of activity limitations such as fatigue and car driving that were reported only in an open-set questionnaire. It also brings in the work-related items that can be severe and impact greatly on the quality of life in MD, as well as specific participation restrictions, such as problems in shopping, doing household work, performing sport activities and gardening [44]. Among personal contextual factors, the restrictions in life and uncertainty are also important [44]. These items were reflected in anxiousness which was one of the most significant factors correlating with the quality of life [32].

In several instruments measuring quality of life such as 15-D, SF-36 as well as in the perception of ‘wellness’ changes in vitality has been reported in MD [45]. About 70 % of the subjects with MD had reduced vitality [46]. Reduction of vitality correlated with increased anxiety, reduction of quality of life and with several items describing participation restrictions. The reduction in vitality seems to be a consequence of the condition (in this case vestibular dysfunction) rather than a causative factor for MD [32, 47, 48]. Although personality trait was associated with anxiety and vitality, the personality trait was regarded as a modifying factor for the condition. The relatively minor role of the personality trait in quality of life and disease-specific impact has been documented earlier [39, 48, 49]. Van Cruissen et al. [47] indicated that the psychological profile of MD patients seems comparable to patients with other chronic conditions.

**Fig. 1:**
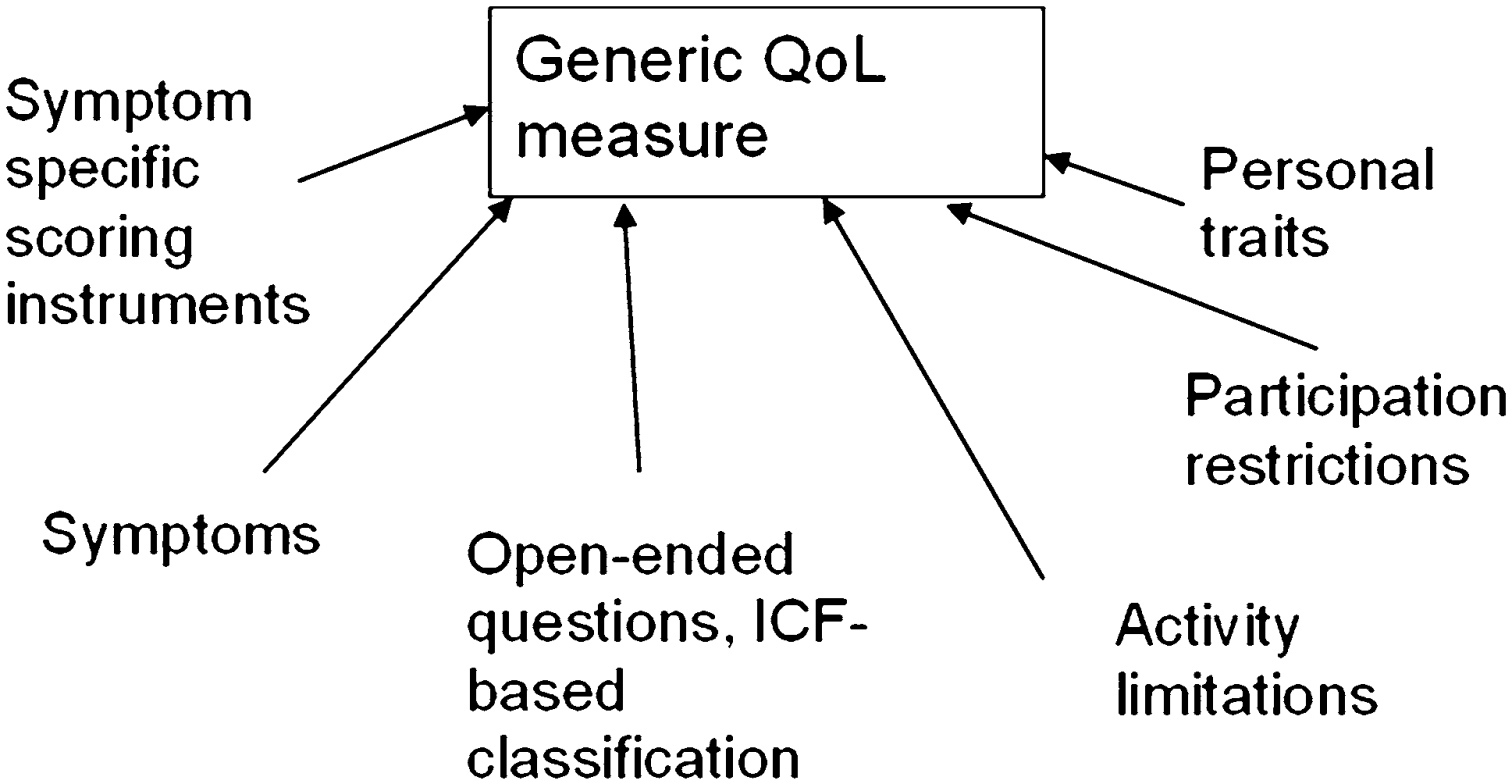
Different approaches used to analyze the impacts of Menière’s Disorder all of which influence generic measures of quality of life (QoL). The disease-specific model can be built from impairments caused by symptoms, open-ended questions, activity limitations or participation restriction (modified from [32]). All these different measures display specific aspects of QoL but are not interchangeable with the outcome of generic QoL instruments

To summarize, MD causes restrictions in a very broad spectrum of personal activities as well as in contextual factors and is characterized by reduced vitality and uncertainty of control of life. The restricted formulation of complaints in current classifications does not explain the individual constraints caused by the illness. The condition may lead to restrictions and limitations that are not directly related to the disease at first glance [44]. There are very few reports in the literature describing the complaints associated with fatigue and especially social isolation [38, 48]. The assumption that healing an impaired function alone would restore the full health in patients with MD is erroneous, since the social participation forms the core construct to achieve any goal-directed behavior [40, 50]. We, therefore, encourage future studies in MD to include the above-mentioned measures of health (Fig. 1), especially vitality and its association with social and personal isolation and to apply holistic therapeutic efforts in MD.

## Evidence from MR imaging in humans

Recent developments of 3 T MR imaging provide a tool for visualizing EH with gadolinium chelate (GdC) as the contrast agent. Following the development of separate visualization of the endo- and perilymphatic compartments by Zou et al. [8], Naganawa et al. [51] and Nakashima et al. [52, 53] developed specific algorithms using Fluid Attenuation Inversion Recovery sequences (FLAIR) that will demonstrate minute amounts of contrast agent in the inner ear [54]. Later, they demonstrated that 3-D recovery turbo spin echo with real reconstruction (3D-real IR) showed higher contrast between the non-enhanced endolymph and the surrounding bone [55]. With the new imaging techniques, EH can be demonstrated in vivo and can confirm the diagnosis. Recently, it has been demonstrated that EH can differently affect cochlear and vestibular compartments and cause different complaints [28]. The value of EH imaging in the differential diagnosis has been shown for the example of patients with clinically suspected vestibular migraine [56]. Furthermore, EH could be demonstrated to progress over time [57] during the disease course, and to be correlated with the deterioration of cochlear, saccular and hSCC function [58–61]. However, the association between clinical symptoms and EH is not uniform in each patient, as hearing can be relatively well preserved despite prominent endolymphatic hydrops. Nakashima et al. [62] and Fiorino et al. [63] have demonstrated, with MRI, that EH was present in all living patients with definite MD.

**Fig. 2:**
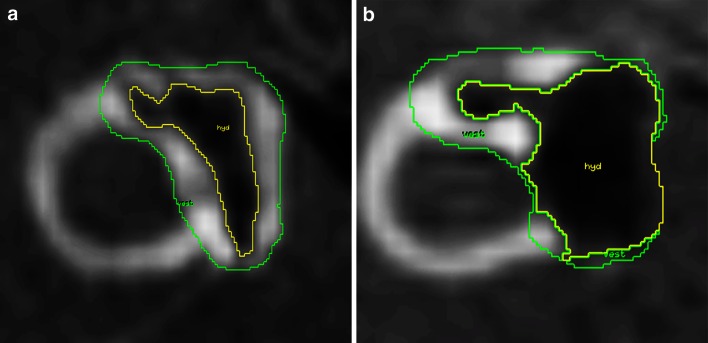
Assessment of vestibular endolymph space in a right inner ear using regions of interest (ROI). The outer ROI defines the cross-sectional area of the vestibulum at the level of the horizontal semicircular canal (“vest”). The inner ROI defines the endolymphatic space inside the vestibulum (“hyd”). **a** The vestibular endolymph ratio in this patient is 0.35, corresponding to mild EH. **b** The vestibular endolymph ratio in this patient is 0.64, corresponding to significant EH (Figure reproduced from [61])

The classification of the degree of endolymphatic hydrops is performed separately for the vestibulum and the cochlea, based on previously documented criteria [64]. The normal limit of ratio of the endolymphatic area over the vestibular fluid space (sum of the endolymphatic and perilymphatic area) is 33 % and any increase in the ratio would be indicative of EH. According to these criteria, *mild EH* in the vestibule covers the ratio of 34–50 % and significant *EH* covers the ratio of more than 50 % in the vestibule. Examples of mild and significant vestibular EH are given in Fig. 2. The respective evaluation of the ratio of the endolymphatic area in the cochlea is correlated to the displacement of Reissner’s membrane. Normally, the Reissner’s membrane remains in situ and is shown as a straight border between the endolymph containing scala media and the perilymph containing scala vestibuli. Mild EH displays an extrusion of the Reissner’s membrane towards the scala vestibuli and results in an area enlargement of the scala media while not exceeding the area of the scala vestibuli. Significant EH causes an increase of the scala media with an area larger than that of the scala vestibuli. Based on previous MRI studies in normal subjects, Nakashima et al. suggested 33 % as the upper limit for the enlargement of endolymphatic space of the vestibule [64]. The normal values that we use have been recently confirmed by other researchers [63, 65].

**Fig. 3:**
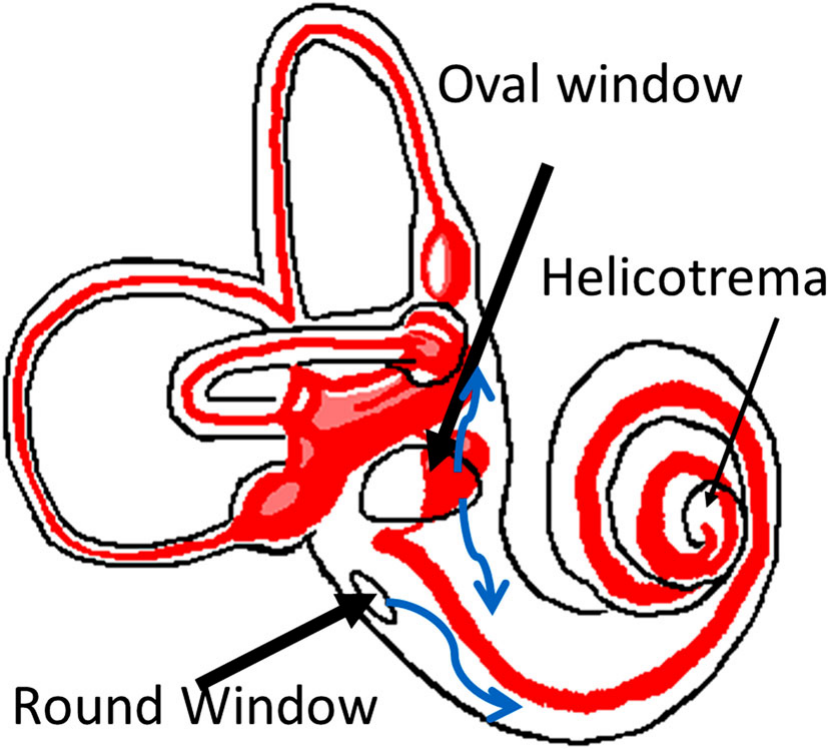
Entry of intratympanically applied drugs into the inner ear perilymph space (*white*) via the round and oval windows. Endolymph space is marked in *red*

For clinical MR imaging of endolymphatic hydrops, two alternative routes of GdC application may be used: intravenous (i.v.) or intratympanic (i.t.). After microscopically controlled application of GdC into the middle ear cavity, it enters the inner ear via the round and oval windows (Fig. 3). The benefit in i.t. delivery is that it achieves higher GdC concentrations—with a significantly lower total administration dosage—than i.v. delivery and the pathology is easier to recognize. However, the i.t. application is off-label, and in our hands about 5–10 % of patients have insufficient GdC uptake from the middle ear. I.t. administration of GdC reduces the risk of systemic toxicity, although it may potentially cause local irritation and toxicity [66, 67]. Current clinical data, however, reveal no evidence of ototoxicity after i.t. application [68–70]. If the clinical presentation suggests a disturbance of the blood–labyrinth barrier, e.g., due to inflammatory processes, this requires i.v. application of GdC to visualize this pathology. In their most recent imaging techniques of the inner ear, Naganawa and Nakashima [70–72] used i.v. administration of GdC with subtraction technique in 3T MRI. With a single dose of i.v. GdC, EH was visualized at 4 h post-injection in humans.

The development of dynamic imaging techniques of the inner ear has provided two important new insights into MD: (1) the cochlear and vestibular compartments can be differently affected. (2) EH is very often present in the “asymptomatic contralateral ears” [28, 53]. It has been well known since long that in typical unilateral MD, the incidence of symptomatic and functional involvement of the contralateral ear increases almost linearly with the length of observation, resulting in bilaterality rate of almost 50 % at 30 years after onset of unilateral MD [92]. Initial clinically bilateral presentations of MD, however, are rare. With the advent of endolymphatic hydrops imaging, we now find that even in clinically unilateral MD, the proportion of contralateral hydropic changes of the inner ear is surprisingly high, and was reported to reach 65 % of clinically “asymptomatic contralateral ears” in an average MD population [28]. This would indicate that MD is a systemic disease. In a recent study, EH was present in 190 out of 205 ears (93 %) with symptoms attributable to MD [28]. Table 1 demonstrates that EH occurs more frequently in the vestibule than the cochlea but most commonly the EH was found in both cochlea and vestibule.

**Table 1:** Endolymphatic hydrops in patients with symptoms associated with Menière’s disorder classified with the AAO-HNS as possible, probable and definite Menière’s disorder (205 ears with symptoms) and also in 45 contralateral ears without symptoms are included

Symptom/diagnosis	EH in cochlea only	EH in vestibule only	EH in both	Total with EH
Possible MD (*n* = 122)	8	43	57	108
Probable MD (*n* = 15)	2	4	8	14
Definite MD (*n* = 68)	1	4	63	68
Total (*n* = 250)	11	51	136	219

Cochlea and vestibule are analyzed separately. Table modified from Pyykko et al. [28]

Of equally great interest are the findings on EH in other disease entities of the inner ear. The great advantage of these imaging data over the autopsy data is the much more detailed clinical description and the perfect temporal association between the EH and the clinical symptoms.

Table 2 summarizes the currently published imaging data on patients that have not been clinically classified as definite MD cases. This emerging new body of evidence allows for some first observations:

**Table 2:** Summary of published reports of EH in patients that were not clinically classified as definite Meniére’s disease

Entity	*N*	With EH (%)	Remarks	References
FLFSNHL	1	1 (100 %)		[73]
	8	6 (80 %)		[74]
	56 ears	38 cochlear EH, 44 vestibular EH	No. of patients with EH not given	[75]
	1	1 (100 %)		[76]
	1	1 (100 %)		[77]
	3	3 (100 %)		[78]
	43	40 (93 %)		[28]
	8	8 (100 %)	All had EH in Cochlea and Vestibulum. The two cases with severe vestibular EH had absent VEMP	[79]
	5	5 (100 %)		[80]
ALFSNHL	1	1 (100 %)		[81]
	2	2 (100 %)	Both had EH in the apical cochlear regions	[82]
RPV	64	31 (48 %)	All patients had horizontal Nystagmus during attacks	[83]
	3	0 (0 %)		[74]
	1	0 (0 %)		[84]
	56	29 cochlear EH, 47 vestibular EH	No. of patients with EH not given	[75]
	2	1 (50 %)		[85]
	2	2(100 %)	EH was more pronounced in Vestibulum in all 3 cases	[78]
	17	15 (88 %)		[28]
SSNHL+V	7	4 (57 %)	Average hearing loss was 90 dB.	[86]
SSNHL	8	2 (25 %)	EH in Cochlea and Vestibulum. MRI at 2 and 11 months after SSNHL. Interpreted as DEH cases	[87]
	4	0 (0 %)		[74]
	1	0 (0 %)	HL was 68 dB	[85]
hSCC malformation	11	9 (82 %)	6 cases had severe EH	[88]
DEH	11	8		[74]
	7	7 (100 %)	Most had EH in both Cochlea and Vestibulum	[89]
	2	2 (100 %)		[82]
	1	1 (100 %)		[85]
	5	5 (100 %)		[90]
	2	2 (100 %)		[80]
VS	13	4 (31 %)	Only the vestibulum could be analyzed	[91]
LVAS	1	1 (100 %)		[85]

*N* number of patients, *FLSNHL* Fluctuating low frequency sensorineural hearing loss, *ALFSNHL* acute low frequency sensorineural hearing loss, *RPV* recurrent peripheral vestibulopathy, *SSNHL*+*V* sudden sensorineural hearing loss with vertigo, *SSNHL* sudden sensorineural hearing loss, *hSCC* horizontal semicircular canal, *DEH* delayed endolymphatic hydrops, *VS* vestibular schwannoma, *LVAS* large vestibular aquaeduct syndrome

The patients with fluctuating low frequency hearing loss very often have EH, and there is a tendency towards more apically located cochlear EH. These are analogous to the “cochlear MD” entity as defined by the AAO-HNS 1972 guidelines. On the other hand, a pure sudden sensorineural hearing loss (not affecting the low frequencies) seems not to be clearly associated with EH. For the other patient groups, with less typical presentations, however, there are two different entities emerging: those with EH and those without EH (Table 3).

**Table 3:** Proposed terminology for inner ear diseases related to endolymphatic hydrops, based on clinical and imaging findings

Proposed new terminology	Old terminology	Other terms
Primary hydropic ear disease (PHED)		
Cochleovestibular type	Definite MD	Typical MD
	SSNHL+V	
Cochlear type	Cochlear MD	FLFSNHL
	ALFSNHL	
Vestibular type	Vestibular MD	RPV, Forme fruste
Secondary hydropic ear disease (SHED)		
Cochlear/vestibular/cochleovestibular type, associated with:	Secondary MD	Menière syndrome
VS		
LVAS		
Labyrinthitis, meningitis		
Noise induced hearing loss		
Trauma		
Congenital hearing loss	DEH	
Inner ear malformation		
…		

*FLSNHL* fluctuating low frequency sensorineural hearing loss, *ALFSNHL* acute low frequency sensorineural hearing loss, *RPV* recurrent peripheral vestibulopathy, *SSNHL*+*V* sudden sensorineural hearing loss with vertigo, *DEH* delayed endolymphatic hydrops, *VS* vestibular schwannoma, *LVAS* large vestibular aquaeduct syndrome

In contrast to the “cochlear MD”, the patients with “vestibular MD” show more variability, but still a significant portion of them has EH. A probable explanation for this observation is the fact that—in contrast to the “cochlear MD” group which is defined by the very specific audiometric finding of fluctuating hearing levels predominantly in the low frequencies—in this “vestibular MD” group there has not yet been identified a distinctive vestibular phenotype. In analogy to the “cochlear MD”, it is possible that a predominantly vestibular EH phenotype could be a certain pattern of abnormalities within the different vestibular function tests. A similar phenomenon linked to EH is well described in definite MD patients: whereas the caloric vestibular response is declining relatively early in the disease course, the vestibuloocular reflex as assessed by the head impulse test is remarkably well preserved until the rather late stages of the disease. This constellation is in stark contrast with, e.g., the entity of vestibular neuritis, where both tests are regularly pathologic. Whether a distinctive vestibular phenotype pattern is also present in “vestibular MD” still remains to be determined. Large-scale studies in this only recently recognized specific clinical and morphological entity are not yet available, but will likely promote our understanding of MD and EH in the future.

## Proposed new terminology based on clinical and imaging findings

Based on the above-mentioned evidence, in order to simplify and clarify the terminology for patients with symptoms formerly described in various ways, e.g., “cochlear MD”, “vestibular MD”, “forme fruste”, “atypical MD”, “monosymptomatic MD”, and in order to enable a description more closely related to the underlying pathology, we propose a new terminology for these clinical entities.

In this system, two main categories of inner ear disease with underlying EH are recognized: Primary Hydropic Ear Disease (PHED) and Secondary Hydropic Ear Disease (SHED). PHED includes not only the definite MD patients, but also the other clinical entities with the clinical phenotype formerly described as “cochlear MD” or “vestibular MD”. The individual symptomatologic differentiation is described by the addition of “cochlear” or “vestibular” or “cochleovestibular type”. This category (PHED) is characterized by the absence of any evident cause for the EH, i.e., a condition or preceding event that is likely to have a significant contribution to the formation of EH. If, in contrast, such a condition, e.g., tumors, malformations, infections, noise or other traumas that affect the inner ear can be identified in the patient, then the second category of SHED should be used. We are aware that high-resolution inner ear imaging is presently not available in all institutions. Therefore, the annotations of “suspected” and “certain” should be used, depending on the confirmation of EH in the individual patient by MR imaging.

Examples would be: “a 45-year-old patient with certain PHED of the vestibular type.” Or “a 20-year-old patient with suspected SHED of the audiovestibular type associated with LVAS”.

Especially for the entity of so-called “recurrent peripheral vestibulopathy”/“vestibular MD”, which is still an only vaguely defined clinical presentation, we expect that the addition of EH to the description of these patients will add important pathological information and help to define the vestibular phenotype of these patients. Furthermore, and even more important for the development of new therapeutic strategies, this proposed new classification may lead to an earlier identification of EH during the disease course, since health practitioners will likely be more aware of EH as the potential underlying pathology in patients that do not (yet) display the full-blown triad of MD symptoms. Therefore, therapeutic interventions may be possible earlier in the disease course, hopefully increasing the chance of halting or even reversing the further progression of EH.

## Conclusion

Recent studies have shown that the description of functional impairments in MD restricted to vertigo, hearing loss and tinnitus as pure symptoms do not sufficiently reflect the wide-ranging impact on quality of life that MD patients are facing. Therefore, personal factors and measures of activity and vitality should be included in future studies.

The milestone development of MR imaging of endolymphatic hydrops supports the central role of endolymphatic hydrops in the pathology of MD, and confirms the same result from temporal bone studies. It has improved the differential diagnosis in suspected MD and warrants the discussion about a new pathology-based description of clinical entities that display various symptoms of inner ear dysfunctions due to endolymphatic hydrops.
